# Intestinal energy harvest is associated with post-bariatric surgery weight loss

**DOI:** 10.21203/rs.3.rs-4031151/v1

**Published:** 2024-03-29

**Authors:** Ian Carroll, Yunzhi Qian, Alicia Sorgen, Kristine Steffen, Leslie Heinberg, Kylie Reed, Aliyah Malazarte, Anthony Fodor

**Affiliations:** University of North Carolina at Chapel Hill; University of North Carolina at Chapel Hill; University of North Carolina, Charlotte; North Dakota State University; Cleveland Clinic; University of North Carolina at Chapel Hill; University of North Carolina at Chapel Hill; University of North Carolina Charlotte

## Abstract

**Background/Objectives:**

Metabolic and bariatric surgery (MBS) is the most effective treatment for severe obesity; however, a significant subset of patients does not achieve expected weight loss or have substantial weight recurrence over time. The intestinal energy harvest is a potential determinant of varying weight loss outcomes, but with limited exploration. We assess the relationships between diet, intestinal energy harvest, and weight outcomes over 24 months in individuals who have undergone MBS.

**Subjects/Methods:**

Calorie absorption was assessed with bomb calorimetry and dietary questionnaires before and after MBS. Within a total of 67 patients, fecal energy density was measured in 67, 56, 60, 67, 44, 47 samples at 0, 1, 6, 12, 18, and 24 months, respectively. Multivariate regression was developed to identify potential weight loss predictors, and random forest algorithms were employed to forecast weight results based on intestinal energy harvest.

**Results:**

Intestinal energy harvest enhanced the predictability of patient weight loss outcomes with random forest models. A notable difference in relative fecal energy content was observed between patients experiencing optimal and sub-optimal weight loss (p<0.01). Prior to MBS, an increased energy content in feces (indicating less energy absorption) is associated with greater weight loss after the operation. Associations between diet and energy harvest were insignificant.

**Conclusion:**

MBS changes energy harvest capacity post-surgery. A higher relative fecal energy content (lower energy absorption) at one month correlates with better weight loss outcomes at 6M, 12M, 18M and 24M post-MBS. Findings may guide the development of diagnostic tools and treatment guidelines for patients at risk of suboptimal weight loss outcomes.

**CLINICAL TRIAL REGISTRATION:**

The trial is registered at clinicaltrials.gov (NCT03065426).

## Introduction

Obesity is a complex chronic disease with a worldwide prevalence that has nearly tripled since 1975(1). Severe obesity is characterized by a body mass index (BMI) > 40, or BMI > 35 with obesity-related health conditions such as inflammation and insulin resistance. The age-standardized prevalence of obesity has increased from 4.6% in 1980 to 14.0% in 2019(2). To date, metabolic and bariatric surgery (MBS), including Roux-en-Y Gastric Bypass (RYGB) and Sleeve Gastrectomy (SG), is increasingly recognized as the most effective treatment to induce body weight loss and metabolic improvement (e.g., enhanced insulin sensitivity, reduced inflammation) in patients with severe obesity(3). RYGB causes food to bypass the duodenum and portion of the jejunum, potentially leading to decreased calorie and nutrient uptake(4). Sleeve gastrectomy removes approximately 80% of the stomach from the greater curvature, reducing food consumption and altering gut peptides associated with hunger(3). Patients lose approximately 30% of their baseline weight after MBS(5); however, a significant subset of post-operative patients do not achieve expected weight loss or have substantial weight recurrence over time. The factors contributing to variations in post-surgical weight loss outcomes remain unclear, and limited attention has been given to controlling variables that may confound weight loss results, such as dietary intake.

Energy harvest is a process by which the human body extracts energy from food in the gut, and individuals have different capacities for intestinal energy harvest. Emerging research suggests that the dynamics of intestinal calorie absorption play a pivotal role in weight loss outcomes among bariatric surgery patients(3,6). Specifically, some patients appear to have a less efficient capacity for intestinal energy harvest following MBS. This inefficiency could lead to a greater excretion of energy rather than its absorption into the body, thereby contributing to more significant weight loss. This mechanism might explain the variability in weight loss outcomes post-MBS and offers a new avenue for personalized treatment plans that take into account an individual’s unique metabolic responses.

There are limited data concerning the longitudinal relationship between dietary consumption and post-MBS weight trajectory(7,8), but the volume of research is limited. Similarly, the connections between dietary intake and energy harvest affecting weight have not been adequately investigated. There is an urgent need to better characterize the combined influence of dietary intake and energy harvest on optimized weight loss outcomes in individuals with obesity prior to and post-MBS. In this paper, we focus on the hypothesis that intestinal energy harvest influences post-operative weight loss among individuals undergoing MBS. To explore this hypothesis, we employed a mixed-methods longitudinal study utilizing stool samples, dietary data, and clinical outcomes from two separate MBS clinics. We hypothesize that there is a link between energy harvest and patient weight management outcome, and we investigate this potential relationship in patients undergoing MBS.

## Materials And Methods

### • Participant recruitment

Data were collected as part of the BioBehavioral Trial, a prospective, 24-month longitudinal study that aims to identify predictors of weight loss trajectory post MBS. Patients were recruited from two geographically distinct clinical sites, and were approved by the Institutional Review Boards at both sites. This NIH-funded study (1RO1 DK112585–01 & 3R01DK112585–01) is registered on ClinicalTrials.gov (Trial ID NCT03065426), and the protocol for the study has been described previously(9). Participants in the pre-operative evaluation process for RYGB and SG were offered the opportunity to volunteer for this study. Participants with informed consent were assessed at baseline (pre-surgery) and specified time points (1, 6, 12, 18, and 24 months) post-MBS. Patient inclusion and exclusion criteria (e.g., current medication known to significantly influence gastrointestinal transit time) were discussed in the previous study(9).

### • Dietary intake questionnaire

The Automated Self-administered 24-hour (ASA24) dietary recall questionnaire was completed by participants pre-surgery and at each time point post-MBS. The web-based system has been used widely in nutrient research and is considered the current gold standard method of assessment of food intake(10,11).

Participants filled out the ASA24 dietary recall over three days at each time point, ideally incorporating at least one weekday and one weekend day— a goal that was achieved approximately 68% of the time throughout the study(7). Macronutrient intake (e.g., total fat, protein, carbohydrate, and fiber) and total energy intake were estimated based on the questionnaire. Participants who had daily total energy intake less than 500 calories or greater than 5000 calories were considered outliers and removed from data analysis.

### • Weight

Patients were weighed in light clothing without shoes at each study visit. Patients were classified into “optimal weight loss” or “sub-optimal weight loss” categories depending on the median percentage of excess weight loss (%EWL) at different time intervals including: 1, 6, 12, 18, and 24 months. Specifically, patients reaching or exceeding the median %EWL at a specific timepoint were deemed to have “optimal weight loss,” while those falling below this median were considered to have “sub-optimal weight loss.”

### • Bomb calorimetry

Fecal samples were collected at baseline and each timepoint post-MBS and stored at −80°C until analysis. Fecal sample energy density was measured via bomb calorimetry using the Parr 6200 Calorimeter with a Parr 6510 water handling system (PARR Instrument Co, Moline, IL, USA). Intestinal energy harvest is estimated using relative energy content (REC), which is calculated using the number of calories in a fecal sample (obtained from bomb calorimetry) divided by the total energy intake by the patient the previous day (obtained from ASA24 dietary questionnaire). Higher fecal REC indicates that less energy was absorbed in the intestine, thus potentially contributing to more weight loss.

### • Statistical analysis

Weight loss outcomes were reported as BMI change from baseline, % Excess weight loss (%EWL), % Total body weight loss, and Total weight loss. The percentage of total body weight loss was calculated for all patients at each timepoint using the difference between initial weight and final weight divided by initial weight. At each time point, patients were grouped into optimal weight loss and sub-optimal weight loss group based on their %EWL at that time interval. Differences in fecal REC between the optimal and sub-optimal weight loss group at all timepoints (1M, 6M, 12M, 18M, and 24M) were compared using Wilcoxon’s rank-sum test.

The associations between the relative energy content rate and dietary intake—- specifically, daily total kilocalories and grams of protein, carbohydrates, fiber, and fats—were evaluated using non-parametric Spearman rank correlations. Multivariable linear regression analyses were implemented to identify factors that have a significant effect on weight loss outcomes among patients post MBS. Statistical analysis was conducted using the Statistical Analysis System (SAS V.9.4, SAS Institute, Cary, NC) with a P-value of 0.05 as the level of significance for statistical tests. Random forest algorithms were developed using the caret package in R(12).

## Results

### • Study population

**Figure 1:**
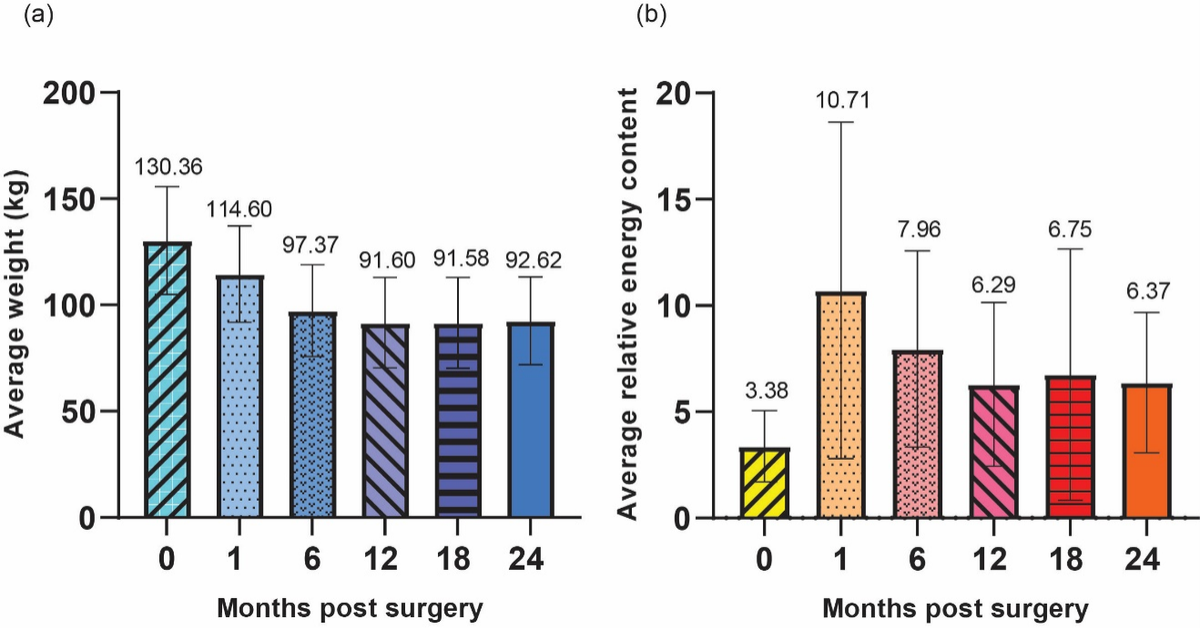
Average weight and relative energy content at each timepoint. (a) Average weight (kg) with standard deviation at each timepoint (b) Average relative energy content with standard deviation at each timepoint.

Among the 67 participants who underwent MBS, met the study inclusion criteria, and provided their consent, 59 (88.06%) and 8 (11.94%) were female and male, respectively. The average age of patients was 41.94 (± 9.28) years. The participant pool was predominantly Caucasian, accounting for 73.13% of the total, with Black or African American individuals making up 20.90%. Patients identifying with more than one race, Hispanic or Latino, and Native Hawaiian/Pacific Islander comprised 2.99%, 1.49% and 1.49%, respectively (**Table 1**). Out of the total number of participants, 41.79% (or 28 individuals) underwent MBS at Clinic 1. Out of these participants, 13 underwent Roux-en-Y Gastric Bypass (RYGB) and 15 underwent sleeve gastrectomy. Clinic 2 accounted for 58.21% (or 39 individuals) of participants. Out of these, 36 participants underwent Roux-en-Y Gastric Bypass surgery while 3 underwent sleeve gastrectomy (**Table 2**). Out of all the 67 patients, fecal energy density was measured in 67 patients at 0M, 56 patients at 1M, 60 patients at 6M, 67 patients at 12M, 44 patients at 18M and 47 patients at 24M. Weight loss outcomes including change in BMI, %EWL, % total body weight loss, and total body weight loss were summarized (**Table 3**). In the first 12 months following surgery, there was a consistent decline in the average weight across all patients. However, the rate of weight loss decelerated thereafter, and by the 24-month mark post-MBS, there was an observed increase in average weight, which is considered expected and normal among post-MBS patients(13,14)(Figure 1a). Following surgery, there was a notable rise in average REC, which then reached its lowest point at the 12-month mark. After that, the average REC slightly increased (Figure 1b). When analyzed by type of surgery, this trend remained consistent. +− Additionally, a significant difference in REC was observed between the gastric bypass group and the sleeve gastrectomy group at the 1-month post-MBS mark (**Figure S1**).

### • Relative fecal energy content is associated with successful weight loss changes post-MBS

**Figure 2:**
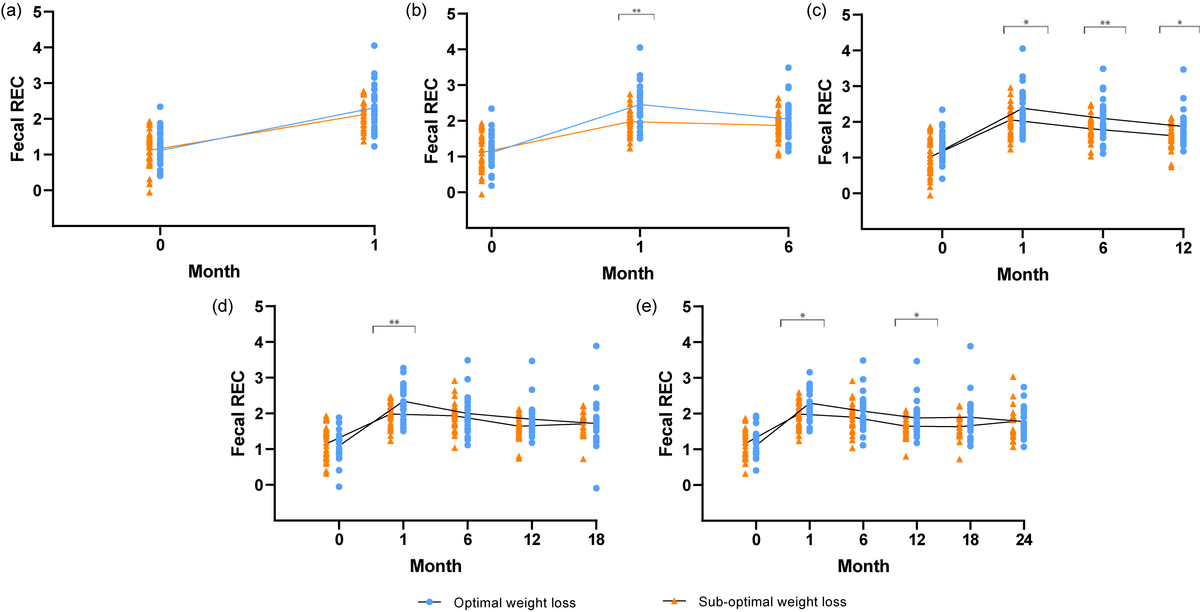
Energy harvest difference between patients with optimal vs. sub-optimal weight changes at five timepoints. (a) Using 1 month optimal vs sub-optimal weight loss grouping (b) Using 6 month optimal vs sub-optimal weight loss grouping (c) Using 12 month optimal vs sub-optimal weight loss grouping. (d) Using 18 month optimal vs sub-optimal weight loss grouping. (e) Using 24 month optimal vs sub-optimal weight loss grouping.

Participants were classified as achieving “optimal weight loss” or “sub-optimal weight loss” post-MBS based on the median %EWL at each time point. The differences in fecal REC between patients that achieved optimal weight loss compared to those that did not were investigated (Figure 2, each point represents the fecal REC value of an individual). Specifically, the fecal REC between patients that achieved optimal weight loss compared to those that did not at one-month post-MBS were compared at baseline and 1-month timepoints (Figure 2a). Similarly, the fecal REC between patients that achieved optimal weight loss compared to those that did not at 6 months post-MBS were compared at baseline,1-month, and 6-month timepoints (Figure 2b). The same number of participants was included in all comparisons (Figure 2a–2e). No significant differences in fecal REC between the optimal and sub-optimal weight loss groups were observed at 1-month post-MBS (Figure 2a). Using 6-month optimal vs. sub-optimal weight loss categories, a significant difference in fecal REC was found between groups at 1 month (p<0.01, Figure 2b) post-MBS—that is, participants at 6 months post-MBS within the optimal weight loss group had a significantly different fecal REC at a prior time point (1 month) to those in the sub-optimal weight loss group. Using 12-month optimal vs. sub-optimal weight loss categories, a significant difference in fecal REC was found between groups at 1 month (p=0.02), 6 months (p=0.01) and 12 months (p=0.02) post-MBS (Figure 2c). Using 18-month optimal vs. sub-optimal weight loss categories, a significant difference in fecal REC was found between groups at 1 month post-MBS (p<0.01, Figure 2d). Using 24-month optimal vs. sub-optimal weight loss categories, a significant difference in fecal REC was found between groups at 1 month (p=0.03) and 12 months post-MBS (p=0.05) (Figure 2e). The p-values remained statistically significant after applying the Benjamini-Hochberg FDR correction for multiple hypothesis testing(15). A supplementary analysis was conducted on participants who had complete fecal REC and weight data at every timepoint (0, 1, 6, 12, 18, and 24M, **Supp. Figure 2**). Among the 28 participants examined, we noted a similar pattern to our initial analysis with the exception of a notable deviation observed at 12M, where no significant distinction between the “optimal” and “sub-optimal” groups was found. It is hypothesized that this discrepancy stems from the relatively small size of the sample (n=28).

Multivariable linear regression models were constructed to identify variables that had a significant effect on the weight loss outcomes among participants who received MBS. Using a stepwise selection method, we identified relative energy content, surgical type, baseline BMI, clinical site, and sex as statistically significant for weight loss outcome for at least one time point (**Table S1**). Specifically, energy harvest from a previous timepoint was selected at the 6-month, 18-month, and 24-month timepoints. To rule out the influence of the type of surgery on differences in energy harvest outcomes, a sub-analysis was conducted where the variable “surgery type” was intentionally kept in the regression models. Besides surgery type, baseline BMI, BMI post-MBS, sex and protein intake were selected as statistically significant factors influencing weight loss outcome for at least one time point. However, energy harvest was not selected in any of the models, implying that the surgery type could significantly impact intestinal energy harvest.

**Figure 3:**
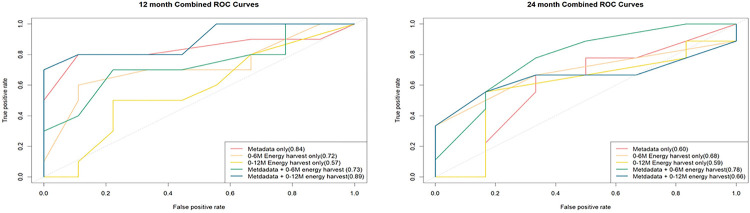
ROC (Receiver operating characteristic) curves for random forest models. The model using energy harvest and metadata as predictors has a higher AUC than the model with only metadata (sex, age, surgery type and clinical site) as the predictor. (a) Using 12-month optimal vs sub-optimal weight loss grouping (b) Using 24-month optimal vs sub-optimal weight loss grouping.

Random forest models were utilized to differentiate between optimal and suboptimal weight loss groups post-MBS, using 5-fold cross validation. Area under the ROC curve (AUC) values were calculated for each prediction model and labeled in Figure 3. In the model for 12 months, the inclusion of energy harvest variables (from 0M, 1M, and 6M) improved the predictive accuracy of the baseline model—which consisted of factors sex, age, surgery type, and clinical site—from 84% to 89%. Similarly, in the 24-month model, the inclusion of energy harvest variables (from 0M, 1M, and 6M) improved the predictive accuracy from 60% to 78% (Figure 3).

### • Absolute fecal energy density shows limited association with macronutrient consumption

We investigated the relationship between absolute fecal energy density and dietary intake of macronutrients, such as proteins, carbohydrates, and lipids, given that these macronutrients contribute to overall caloric intake, which in turn is a factor in estimating fecal REC. After applying the Benjamini-Hochberg FDR correction, Spearman correlation tests revealed no significant correlation between macronutrient consumption and absolute fecal energy density (**Table 4**). These findings suggest that the influence of macronutrient consumption on fecal energy density measurements is relatively minimal.

## Discussion

To further elucidate the role of intestinal energy harvest in post-MBS weight loss outcomes, we sought to examine associations between short- and long-term intestinal relative energy content with weight changes among 67 post-MBS participants for 24 months. Our study finds that higher fecal REC levels correspond with reduced energy absorption in the gut, which can be instrumental in promoting weight loss after surgery. This correlation was notably significant at the 6-month (Fig 2b), 12-month (Fig 2c), 18-month (Fig 2d), and 24-month (Fig 2e) post-MBS timepoints (Fig. 2b), but less so at 1 months (Fig. 2a). At all the timepoints (1,6,12,18, and 24M), fecal REC levels were higher (less energy absorption) in the optimal weight loss group after the baseline (0 month) timepoint even when this relationship was not statistically significant. These results are intriguing in that they suggest that the patient population that responds more successfully to surgery extracts less energy from the gut. These results are pivotal in providing evidence for predicting personalized intestinal energy harvest responses in weight loss management(16).

The fecal REC levels, as a proxy of gut energy harvest capacity, can also act as predictive indicators, helping to classify participants into optimal or suboptimal weight loss categories. Incorporating energy harvest from a previous timepoint into the random forest model enhanced its ability to predict weight loss outcomes at 12- and 24-month post-MBS (Fig. 3). The substantial difference in REC between the groups experiencing optimal and sub-optimal weight loss has important clinical implications. It has the potential to enable clinicians to identify, as early as one-month post-MBS, those patients likely to experience less than optimal weight loss outcomes. From a prognostic standpoint, variations in intestinal energy harvest could potentially predict the extent of post-MBS weight loss, with elevated fecal REC possibly linked to optimal weight loss results. On the therapeutic front, the identification of an unfavorable energy absorption profile may guide interventions, such as customized lifestyle changing plans, aimed at maximizing post-surgical weight loss outcomes. Therefore, integrating insights into gut energy absorption capabilities with bariatric surgical procedures can enhance the precision and comprehensiveness of obesity treatment strategies.

We examined the impact of fecal energy density on weight loss outcomes through multivariable regression analyses. Interestingly, when we ensured the surgical type remained constant in the model, the energy harvest from both baseline and 1M ceased to be selected in models forecasting the % total body weight loss at the 18- and 24-month post-MBS. Earlier research has highlighted varying outcomes from different MBS procedures, specifically when comparing sleeve gastrectomy to gastric bypass(17,18). This divergence in outcomes hints at the possibility that the type of surgery could significantly modulate the intestine’s energy harvesting capability. Additionally, it suggests that the efficacy of these surgeries, particularly the Roux-en-Y gastric bypass (RYGB), may be partially attributed to its influence on energy extraction processes. Different surgical procedures may alter the gut’s anatomy and functionality in distinct ways, potentially affecting its capacity to extract energy from food.

We also investigated the link between fecal energy density and macronutrient consumption. We concluded that the association between macronutrient consumption (carbohydrate, fat, protein, fiber) and fecal energy density is insignificant. Previously, Kanerva et al. found that individuals who prioritized protein and carbohydrates over fat, and those who preferred protein to carbohydrates, experienced more substantial weight loss(19). While Krebs et al. reported that weight loss is linked to higher protein and lower carbohydrate intake(20). The inconsistency in findings might stem from variations in follow-up durations, sample counts, dietary intake estimation methods, and the extent of underreporting. These controversial findings underscore the need for further research to delve into diet composition and its relation to weight results.

Informed by previous research on gut microbiota, it becomes clear that the gut microbial communities have a notable impact on intestinal energy harvest(21,22). This is in line with our findings that demonstrate a correlation between gut energy harvest and weight loss following bariatric surgery. Considering our established data on the composition of the gut microbiota in these post-MBS patients(23), a logical next step would be to deepen our investigation into how these microbial compositions directly influence energy harvest and, consequently, weight loss outcomes. Such an inquiry will bridge our current understanding of the relationship between bariatric surgery and weight loss with the intricate role played by the gut microbiota. This research direction could offer valuable insights into optimizing post-surgical outcomes and tailoring post-operative nutritional guidelines for patients.

As the original BioBehavorial trial was not an impatient feeding trial, we were unable to collect data that provide more accurate estimates of intestinal energy harvest clinical measurements that may influence our analysis. Specifically, we were unable to collect information on fecal sample weight, number of fecal samples produced daily, intestinal transit time, and resting or total energy expenditure. Although we utilized a dietary assessment developed by NIH and considered a gold standard for self-reported nutritional intake, any measure of dietary recall may potentially lead to inaccuracy in energy intake were not measured. However, the approach we used enabled us to amass a larger number of fecal samples compared to an approach where we implemented a stringent inpatient feeding trial. We also used fecal REC as a reliable measure for gut energy absorption, and our findings consistent with prior research(22,24). This underscores that fecal relative energy content serves as a valid approximation for intestinal energy absorption. However, future research should validate intestinal energy harvest in strictly controlled conditions such as using a metabolic chamber to further elucidate the relationships found in this study.

## Conclusions

In summary, the study offers some of the initial evidence indicating that fecal energy density, a proxy for intestinal energy harvest at previous timepoints, is predictive of future weight loss outcomes post bariatric surgery. Absolute fecal energy density shows limited association with macronutrients consumption. Additional studies are necessary to determine if intestinal energy absorption can consistently predict future weight loss outcomes in patients who have undergone bariatric surgery.

## Data Availability

Data will be available to the extent the NIH requires and is permitted by appropriate laws and rules. Deidentified data will be made available for the journal upon request.
